# Understanding the impact of the economic crisis on child health: the case of Spain

**DOI:** 10.1186/s12939-015-0236-1

**Published:** 2015-10-14

**Authors:** Luis Rajmil, Arjumand Siddiqi, David Taylor-Robinson, Nick Spencer

**Affiliations:** Agència de Qualitat i Avaluació Sanitàries de Catalunya, Roc Boronat 81-95 2nd Floor, Barcelona, 08005 Spain; IMIM (Institut Hospital del Mar de Recerca Biomèdica) Barcelona, Barcelona, Spain; Centro de Investigación Epidemiológica en Red de Epidemiolgìa y Salud Pública CIBERESP, Madrid, Spain; Divisions of Epidemiology and Social and Behavioral Sciences, Dalla Lana School of Public Health, University of Toronto, Ontario, Canada; Department of Health Behavior, Gillings School of Global Public Health, University of North Carolina, Chapel Hill, USA; Clinical Senior Lecturer Department of Public Health and Policy, Whelan Building, University of Liverpool, Liverpool, UK; Emeritus Professor of Child Health, Division of Mental Health and Wellbeing, Warwick Medical School, University of Warwick, Coventry, CV4 9JD UK; 86, Leicester Street, Leamington Spa, CV32 4 TB UK

## Abstract

**Introduction:**

The objectives of the study were to explore the effect of the economic crisis on child health using Spain as a case study, and to document and assess the policies implemented in response to the crisis in this context.

**Methods:**

Serial cross-sectional data from Eurostat, the Spanish Health Interview Survey, and the database of childhood hospitalisation were analysed to explore impacts on child health, and key determinants of child health. A content analysis of National data sources/government legislation, and Spanish literature was used to describe policies implemented following the crisis.

**Results:**

Unemployment rates in the general population (8.7 % in 2005 and 25.6 % in 2013), and children living in unemployed families (5.6 % and 13.8 %) increased in the study period. The percentage of children living under the poverty line, and income inequalities increased 15–20 % from 2005 to 2012. Severe material deprivation rate has worsened in families with Primary Education, while the number of families attending Non-Governmental Organisations has increased. An impact on children’s health at the general population level has not currently been detected; however an impact on general health, mental health and use of healthcare services was found in vulnerable groups. Investment in social protection and public policy for children showed a reduction as part of austerity measures taken by the Spanish governments.

**Conclusions:**

Despite the impact on social determinants, a short-term impact on child health has been detected only in specific vulnerable groups. The findings suggest the need to urgently protect vulnerable groups of children from the impact of austerity.

## Introduction

Understanding the health impact of the Great recession on specific vulnerable groups such as children is important to inform policies at national and international levels. The current crisis has affected the whole European economy, but the potential impact on health in each country depends on several factors including the starting point; mechanisms of social protection and social transfers; and the measures adopted by governments to deal with the crisis [1].

Three phases of the crisis have been described [2]. The first wave (economic impact) was characterised by job losses and reduced household incomes in many countries. The second wave (social impact) was characterised by a high levels of unemployment particularly affecting younger people, increasing the numbers neither in employment nor in education or training (NEETs). The third wave (unequal recovery), that authorities and some media are claiming started in 2014, has been characterized by a slow, uneven return of growth to trend with some areas recovering quickly but other areas remaining in recession.

Spain has been hit hard by the Great recession [3]. The Welfare state in Spain was created more recently than in other western European countries, after the period of dictatorship. The Spanish society and economy inherited a protected and conservative financial sector, an insufficient and regressive fiscal system, and scarcity of social protection and benefits. Consequently, the pre-crisis welfare state was less comprehensive than in other European countries [4]. In this situation any reduction of the welfare state is likely to result in even weaker social protection than in other contexts [5, 6].

Although there are many potential differences between countries, recessions pose risks to health of the general population [7]. Mental health problems, infectious diseases and suicides are becoming more common in countries affected by the economic crisis [8].

It is universally recognised that children represent a particularly vulnerable population group. Inequalities in early child development have been identified as a major contributing factor to inequalities in adult health, depending on the balance of adverse exposures and protective factors in early life [9, 10]. Few studies have been published to date on the impact of the current crisis on children’s health [11, 12] and the responses of specific governments [7], particularly in terms of the potentially mitigating or harmful effects of public policies affecting family economic security and social conditions [13]. The objectives of the present work were therefore to explore effect of the crisis on child health using Spain as a case study, and to document and assess the policies that have been implemented in response to the crisis in this context.

## Methods

A descriptive and exploratory study was conducted using mixed-methods approach. Routinely available data pre-and post crisis was analysed to monitor social determinants of child health. Periodic Spanish National Health Interview Survey (NHIS) and the Minimum Data Set of Hospital Discharge (MDHD) was used to check changes in health behaviours and mental health indicators during the study period, and synthesis of data on key policies that have influenced families with children was analysed to describe government responses. We sought to analyse trends in key social determinants affecting children (poverty and material deprivation); and child health outcomes. A content analysis of the data sources on legislation and a recent supplement published by the Spanish Society of Public Health [3] was used to describe austerity measures. Where possible we sought to identify any differential effects of policies on the basis of socioeconomic status, to test the hypothesis that more vulnerable groups have been disproportionately affected by the crisis, and some of the policy responses in Spain.

### Source of data and variables

#### Key social determinants of child health

Serial cross-sectional databases from Eurostat, OECD, and the Spanish National Institute of Statistics (http://www.ine.es/en/welcome.shtml) were analysed to describe unemployment, child poverty, material deprivation, and measures of income inequality. The source of these data was the Economically Active Population Survey (EAPS) that is a quarterly continuous research focusing on families, whose main purpose is obtaining data on workforce and its several categories (employed, unemployed), as well as on population out of the labour force (economically inactive population). The initial sample is about 65,000 families per quarter, which equals approximately 180,000 persons. Annually data since 2005 to the latest available data was included in the analysis.

Unemployment rate was analysed at the general population level and in the population younger than 25 years, as well as the rate of young people living in unemployed families. Child poverty (%) was defined as the percentage of children living in households with income below 60 % of the median. Indicator of material deprivation was the percentage of children under 17y with unmet basic needs according to the European Union- Survey of Income and Living conditions (EU-SILC, coming from the same registries), stratified by family level of education (Table 1). Income inequality was assessed by the quintile share ratio S80/S20 that puts the income of the top 20 % of the population in relation to that of the bottom 20 %.

**Table 1:** Material deprivation according to the European Union-Survey of Income and Living conditions (EU-SILC)

The definition of material deprivation was based on a selection of items that are considered to be necessary or desirable, namely: having arrears on mortgage or rent payments, utility bills, hire purchase instalments or other loan payments; not being able to afford one week’s annual holiday away from home; not being able to afford a meal with meat, chicken, fish (or vegetarian equivalent) every second day; not being able to face unexpected financial expenses; not being able to buy a telephone (including mobile phone); not being able to buy a colour television; not being able to buy a washing machine; not being able to buy a car; or not being able to afford heating to keep the house warm. The severe material deprivation rate was defined as the proportion of persons who cannot afford to pay for at least four out of the nine items specified above.	

Specific reports on vulnerable families looking for help from Non-Governmental Organisations (NGO) such as Caritas [14] were analysed to assess if austerity measures instituted affect vulnerable and low income families more than those on higher incomes.

#### Population health impacts on families and children

Variables collected to analyse the impact on child health were focused on nutrition habits and violence against children, informed by the results of a previous systematic review [11].

The NHIS is a Spanish nationally representative health interview survey conducted every 4 to 6 years on behalf of the Spanish Ministry of Health (http://www.msssi.gob.es/estadEstudios/estadisticas/encuestaNacional/ense.htm). The sample is independent and representative of each autonomous community (an organizational division of the Spanish territory). The present study included the last two surveys, that were conducted in 2006/07 (pre-crisis) and 2011/12 (after started the crisis). Both surveys were a multistage stratified sample. Within each household with children or adolescents (aged 0–15 years), one child was randomly selected for the children’s questionnaire that was administered to a proxy-respondent (mainly mothers). Study sample was *n* = 6838, and *n* = 4595 in the NHIS 2006/07 and 2011/12, respectively. Variables analysed were general health, mental health, and specific behaviours such as not having breakfast before leaving home, in a period before and after the start of the crisis. Mental health was analyzed by means of the parent version of the Strengths and Difficulties Questionnaire (SDQ, www.sdq.org) administered to the NHIS sample of 4–15 year old children. The range of scores in this scale is 0 to 40, and higher score means worse mental health.

The MDHD database on childhood hospitalisation from the Spanish Ministry of Health (https://www.msssi.gob.es/) due to unintentional injuries (ICD-9 Group 17) and maltreatment (ICD-9 codes 995.50 to 995.59, and 301.51) were analysed for the period 2000–2012. Hospitalisation rates were computed for each year taking into account the number of children hospitalised and census data as denominator.

#### Impacts on vulnerable groups

The data from a study on vulnerable families affected by eviction or at risk of eviction [15] was compared with the NHS 2011–12 to analyse the impact on vulnerable families with children. This data was collected during 2012 and included 177 families with children participating in the study.

#### Policies implemented in Spain since the crisis

National data sources/government legislation and Spanish literature on the institution of economic and policy measures were used to analyse the measures taken by the Spanish government, based on a previous review [16], with a focus on those measures with impacts on families and children. A narrative review of the content was carried out attempting to assess the potential impact of each measure specifically on poor and vulnerable families or at the general population level.

### Statistical analysis

We present trends in cross-sectional data on unemployment, child poverty, material deprivation and income inequalities. Analysis of repeated cross-sectional data by means of joint regression [17] was used to assess trends in hospitalisation during the study period. Joint regression assesses the existence of a significant change of trend in each year and to quantify the annual percentage change (APC) and its statistical significance. The level of statistical significance was established at *p* <0.05.

All procedures were carried out following the data protection requirements of the European Parliament (Directive 95/46/EC of the European Parliament and of the Council of 24 October 1995 on the protection of individuals with regard to the processing of personal data and on the free movement of such data). The ethical and legal requirements in Spain were also adhered to.

## Results

### Impacts on social determinants of child health

**Fig. 1:**
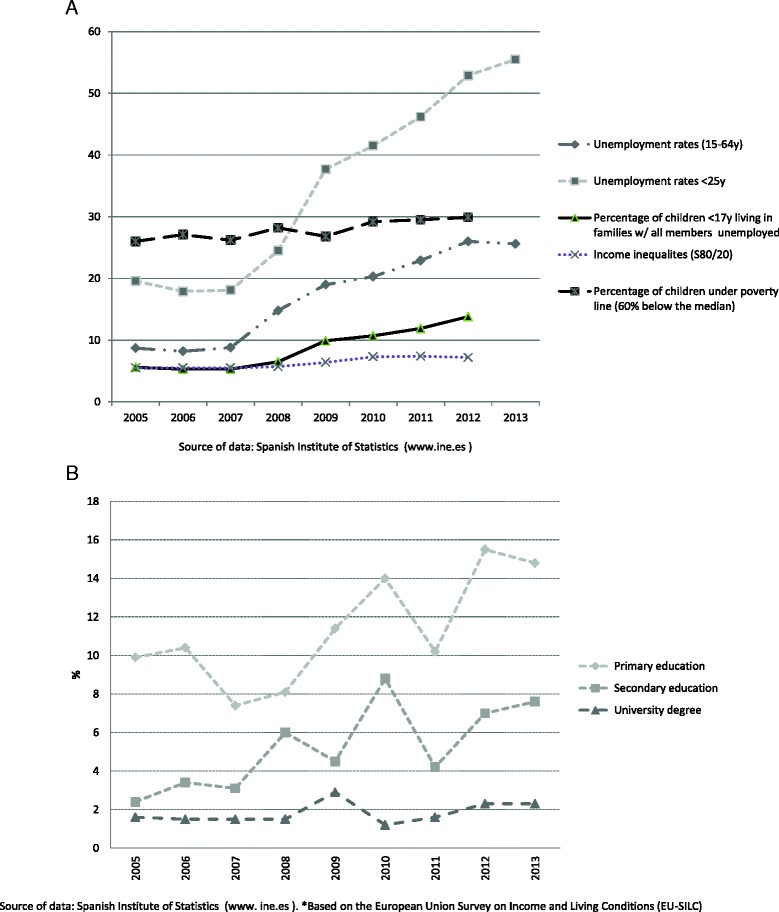
**a.** Unemployment, child poverty and income inequalities. Spain 2005–2013. **b.** Unmet basic needs*. Spain 2005–2013

Unemployment rates in the general economically active population increased from 8.7 % in 2005 to 25.6 % in 2013 (Fig. 1a). Unemployment in the population younger than 25 years increased from 19.6 % in 2005 to 55.5 % in 2013, and the figures for children living in unemployed families increased from 5.6 to 13.8 % in the study period. Income inequalities increased 20 % during the period 2005–2013, and the percentage of children living under the poverty line increased 15 % from 2005 to 2012. Severe material deprivation rate according to parental level of education increased in families with Primary Education from 9.9 % in 2005 to near 15 % in 2013, and from 1.6 to 2.3 % in families with University degree (Fig. 1b).

Some NGO such as the Food bank federation assisted 700,000 beneficiaries in 2007 and 1,5 million people in 2012, most of them families with children. The Caritas report notes that in 2007, 370,251 people attended and in 2011 this figure was 1,015,276. Four percent of the Spanish population lacks resources to meet their basic daily food. A specific aspect of the impact of the crisis on infant feeding has to do with school meals. School meals have become unaffordable for many families and this might be associated with nutritionally poorer diets (Table 2).

**Table 2:** Impact on social determinants, child health outcomes, and on vulnerable population

	Indicators	Results (summary)	Potential impact
Determinants of child health			
Food insecurity and nutrition	NGO reports and reviews on the use of food banks [29]	Exponential increase on vulnerable families looking for help attending basic food needs	Increased inequalities on the risk of undernutrition, obesity and other related conditions. Great impact on vulnerable families
Child health outcomes			
General health	Comparison of the % of fair/poor perceived health in 2006 and 2012 (NHS)	Improvement of perceived health (11 % poor health in 2006 and 6.8 % in 2012) in population 0–14y. No changes in social inequalities detected	No impact during the period analysed
Children’s mental health	SDQ administered to parents of a representative sample of children in 2006 and 2012 (NHS)	Improvement of mean total difficulties score of SDQ, remaining similar inequalities by social class; worse mental health in children from unemployed families comparing two cross-sectional surveys	No impact during the period analysed
Vulnerable population			
General health, mental health, health habits	General health, mental health, not having breakfast before leaving home in a group of 177 children attending Caritas [15]	22.7 % (boys) and 22.9 % (girls) reported poor health (direct emergency attention group) compared to 6.8 and 6.9 % in the general population. SDQ: probable case 61.3 and 37.5 % (boys and girls) vs 9.2 & 7.6 % in the general population. Similar results for not having breaksfast (15.9 & 26.2 % vs 0.1 and 1 %)	A great impact on health, mental health and health behaviours was found in these vulnerable groups
Access and use of healthcare			
	The population of children 0–18y theoretically continuous with universal coverage and no barriers to access	There were at least 14 cases of children in which barriers to access were registered. Many unreported cases of fear of parents due to their irregular administrative situation [30]	Great impact on vulnerable children

*NHS* National Health Survey, *APC* Annual percentage of change, *SDQ* Strengths and Difficulties Questionnaire

### Health impacts

#### General health, family and childhood mental health

Perceived health as poor according the NHIS improved from 11 % in 2006 to 6.8 % in 2012 (Table 2). Results of mental health of children from the NHIS show that the total difficulties score of the SDQ was lower (better) in 2012 compared to 2006 for the total population, with slightly worse scores for those children with all family members unemployed.

#### Unintentional injuries and child maltreatment

**Fig. 2:**
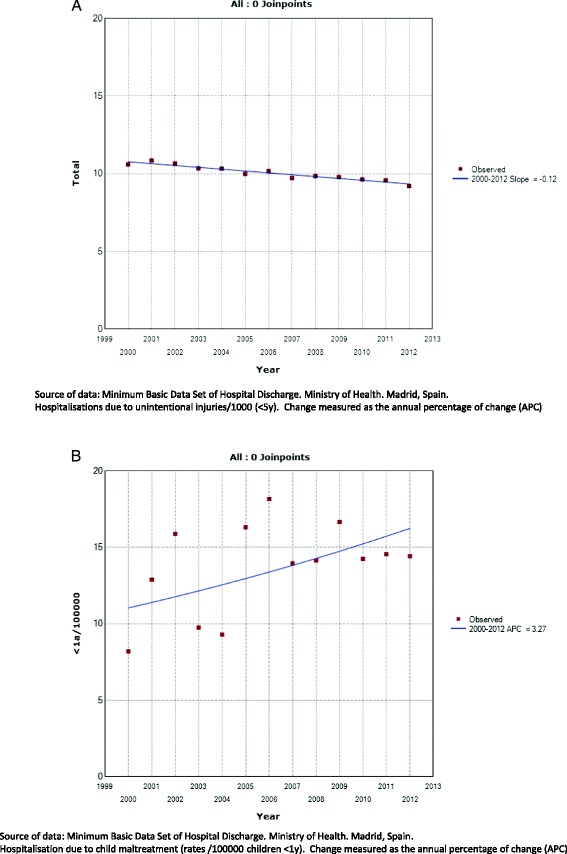
**a.** Hospitalisations due to unintentional injuries in children younger than 5y. Joint regression 2000–2012. **b.** Hospitalisations due to child maltreatment in children <1y. Joint regression 2000–2012

Hospitalisations due to unintentional injuries showed an improvement with an annual percentage of change (APC) of −0.12 in the population <5y (with approximately 10,000 hospitalisations/year) (Fig. 2a). No changes were found in trends of hospitalisations due to maltreatment. The APC was 0.23 from 2000 to 2013, with rates of approximately 10/100,000 in children younger <1y (Fig. 2b).

### Health of specific vulnerable populations

There were 177 children in the vulnerable group attending Caritas, one subgroup required the Direct Emergency Attention due to the need for immediate re-housing and another subgroup of Housing Mediation Services that needed help to negotiate their debt (Table 2). Fair or poor health was higher in the direct emergency attention, and the housing mediation service groups. The probability of suffering a mental health problem was more than 10 times in the group of boys in greater need and 5 times higher in girls, and also similar differences were found in not having breakfast, comparing with data from the NHIS.

### Policies implemented in Spain since the crisis

Table 3 shows the laws and regulations implemented by successive Spanish governments following the crisis, with commentary on the potential impacts on children and families. The main structural and budgetary changes in Spain started in 2010 with an energetic policy of fiscal stability, the fundamental savings of which would be in the expenditure side (austerity) rather than the income. Successive governments used the law via decree enacting a series of initial austerity measures such as reducing wages and the Spanish Constitution was reformed to give prominence to the budgetary stability over other commitments, in accordance with the requirements of the European Union.

**Table 3:** Policies implemented in Spain since the crisis^a^

Measure	Type of measure^b^	Content of measures	Potential impact on family and children
Royal decree law 8/2010 on exceptional measures to reduce public spending (May, 2010)	B	Removal of universal benefits for each birth or adoption (2500 Euros), among other measures of reduction on public spending	The birth benefit started in 2007 (recently); its removal has an impact on the whole society with likely greater impact on poor families
Modification of Article 135 of the Spanish Constitution (September, 2011)	S	Established the primacy of fiscal stability. All governments should respect the structural deficits, and public debt should be a priority in payment	The fact that future budgets were conditioned to pay the debt before attending to the needs of citizens has potential impacts on the whole society
Royal decree law 16/2012 and further developments	S & B	Transform the healthcare system with almost universal coverage to a social security system, and establish the exclusion of specific groups such as undocumented migrants. Increase in co-payments for drugs and also extended it to other sanitary products and services	Theoretically these measures do not apply to children under 18y (they continuous having universal coverage), but the policy particularly impacts vulnerable families and migrant families. The removal of universal coverage has the potential to greatly increase health inequalities.
Spanish general budget 2011 (Dec 2011); law 2/2012 (April 2012); decree law 21/2012 (July 2012); and subsequent laws on the general budget	B	Budget cuts of approximately 20–25 % on public spending; plus other measures addressed to reduce unemployment benefits, and increase VAT from 18 to 21 %	Linear cuts, reduction of unemployment benefits which penalise long term unemployed, and increase in none progressive taxes. All measures have a greater impact on poor families
Stability Programme 2013–16 (April 2013)	S & B	Control of deficit (6.3 % in 2013, and proposal of 5.5 % in 2014, 4.1 % in 2015 and 2.7 % in 2016).	Unknown impact in the short term but the impact could be greater on poor families if the control of deficit is centred on budget cuts
Investment in social protection for children and families	B	Comparing the years 2007, 2010 and 2013 in constant Euros, the latter data showed a reduction of 6.8 % over 2007 and 14.6 % since 2010 [31]. The budget cuts in public education have affected child pre-schooling, among other sectors	Impact on social inequalities and greater impact on vulnerable families
Housing policy: low social protection and a system (pre-crisis) that perpetuate debts even after evictions	S	Evicted families and/or with difficulties in maintaining their houses with precarious jobs or without jobs increased with the crisis	A great impact on vulnerable families and social movements demanding changes in these laws that penalise vulnerable families

^a^Based on the reference [16] for more information see tables 2 and 3 of the mentioned reference; except reference [31] ^b^S: Structural; B: budgetary

Most measures were aimed at reducing spending (austerity) with no attempt to ensure social protection for children. In a previous analysis comparing investments in social protection for children in the years 2007, 2010 and 2013 in constant Euros, the latter data showed a reduction of 6.8 % over 2007 and 14.6 % since 2010. Investment in public policies for children in Spain was 1.4 % of GDP the year 2012 vs. 2.2 % in the EU28 in 2011. The budget cuts in public education have affected child pre-schooling, among other sectors. Even before the crisis, those families evicted from their houses were still liable for mortgage debts. This policy has negative impact on evicted families and/or families with difficulties in maintaining their houses with precarious jobs or without jobs. The number of evicted families has doubled between 2007 and 2012.

#### Access and use of healthcare services

Although the population of children 0–18y theoretically continues to have universal healthcare coverage by law, some cases of barriers to access were detected and also a variability in the implementation of recent policy measures, with a great potential impact on vulnerable children. Moreover, the breaking of universal healthcare coverage in the adult population was also related to cases of healthcare exclusion in children who were refused treatment by hospitals, and parental fear of having to pay for the visit (Table 2).

## Discussion

The study results show a significant deterioration in the social determinants of child health since the crisis and over the period during which austerity measures have been implemented, with increasing social inequality and child poverty. We do not find any immediate effect on child health at general population level but there is evidence of an impact on vulnerable groups.

Among the limitations of the present study should be mentioned the lack of updated and disaggregated data on child population to study the impact on small areas or vulnerable groups. Moreover, the analysis of population average indicators can mask inequalities in specific population subgroups. Secondly, there are difficulties in establishing a causal association between austerity measures and health outcomes. Available data do not allow analysis of the association between social determinants and health outcomes because these aggregate data come from different sources and cannot be easily combined. Further studies using small area or individual level data are recommended to address these questions. Furthermore, longer term follow-up is required to assess the plausible long-run impacts of a deterioration in the social determinants of child health.

The budget cuts in public education have affected the possibility of promoting a more equitable growth and development and early socialisation of children have been reduced in areas with greater economic deprivation [18]. The budget constraint has also affected school canteens. This fact is associated with increased difficulty in providing adequate food especially to the most vulnerable groups, and possibly perpetuates existing inequalities in childhood obesity according to family education level [12, 19].

Difficulties to maintain housing have deteriorated alarmingly, either to pay the mortgage or rent for families with scarce resources. The Spanish laws before the start of the current crisis unfairly penalised those who cannot pay their mortgages. This fact has been worsened by rising unemployment and precarious working conditions of many families due to the economic crisis. Moreover, the increase in the price of energy has increased the difficulties of families to meet their basic needs for water, electricity, heating, etc. These factors have shown a large negative impact on child health of these vulnerable groups [15].

Spain is one of the developed countries with highest increase in the percentage of children at risk of poverty [20]. One in three children in Spain lives at risk of poverty, according to the latest available data. In the face of cuts to welfare support, NGOs have seen a dramatic increase in demand for services since the onset of the economic crisis. Thus, NGOs have tripled the number of families attended since the beginning of the crisis by problems of housing, food or social support while public investment in child protection has declined [21, 22]. Romania, Spain, Bulgaria, Greece and Italy share the highest rates on child poverty and low impact of family and childhood benefits. Moreover, it has been shown a greater impact in children compared with other general population groups [18], similar to what happens in other countries [23, 24].

Social determinants of child health have deteriorated and social inequalities affecting children have increased in Spain with the economic crisis, a fact known to be associated with worse health outcomes in the medium and long term, according to the evidence from previous studies [25]. As part of the response to the crisis, successive Spanish governments have established austerity measures and structural changes in labour protection, as well as in social and health care systems [26]. These measures are affecting families with fewer economic resources, families with all members unemployed, long-term unemployed families, and single parent unemployed families.

The childhood population under 18 years old legally continues with universal access to healthcare services after the promulgation of Decree Law 16/2012, which breaks the universality of access and changes the paradigm of the Spanish healthcare system [27]. However, some cases of healthcare exclusion in children have been reported and variability in the application of the above mentioned decree has been found. Some regions of Spain continue with the previous system without barriers to access while in other regions the decree is variably applied. These barriers to population subgroups, such as migrants in an irregular situation, are associated with an indirect impact on the child population and insecurity in families in these conditions, which are usually those who need more support and access to preventive measures and healthcare services. Likewise, the increase in copayments generates greater difficulty in the most vulnerable groups. The results of the present study are consistent with those of another study that analysed the austerity measures in Europe and showed that they can exacerbate the short-term public health effect of economic crisis [7].

The finding that, despite the impact of austerity in Spain on the social determinants of child health, a short-term impact on child health has been detected only in specific population subgroups and not in the general child population is counter-intuitive and challenges the hypothesis that austerity is detrimental to the health of child populations. Previous studies found poor mental health in the adult population [8] with an increase of 19 % in the percentage of mood disorders, 8 % in anxiety, and 5 % in alcohol abuse after the crisis started, and these increases were associated with unemployment and housing problems. Increased incidence of suicide attempts in the general population was also found [28]. We have not detected changes in children’s mental health trends nor an increase in child maltreatment, effects that were found in a systematic review of the impact of the current crisis in child health [11]. It is likely that, besides the scarcity of data, families play a protective role in Spain, maybe greater than in other contexts. It could be that families take a palliative and resilient role against the negative effects of the crisis. However, if family stress becomes chronic it can cause depletion of family resources. Moreover, according to the conceptual model and the available evidence, the effect of worsening social inequalities in childhood has consequences for the distribution of health in adulthood [8], and specific changes in law that counteract growing income inequalities through the tax, labour, and welfare systems could have sizable benefits for population health and health disparities.

In conclusion, this study aimed to summarise the evidence about the impact of the economic crisis and austerity measures on social determinants and child health. The impact of austerity policies has likely increased child poverty and deprivation with likely effects on child health especially among vulnerable groups. The findings of this case study suggest the need to urgently protect vulnerable groups of children from the impact of austerity.
